# The diagnostic potential of extracellular vesicles in virus-related diseases

**DOI:** 10.3389/fcimb.2025.1641405

**Published:** 2025-07-30

**Authors:** Qing Gao, Yuqing Zhan, Jianhao Zhang, Dongyu Sun, Huayuan Xiang, Chenxuan Bao, Qianqian Gao, Mengyu Zhang, Jianjun Wang, Lingxiang Mao

**Affiliations:** ^1^ Department of Laboratory Medicine, Affiliated Kunshan Hospital of Jiangsu University, Kunshan, Jiangsu, China; ^2^ Jiangsu Key Laboratory of Medical Science and Laboratory Medicine, School of Medicine, Jiangsu university, Zhenjiang, Jiangsu, China

**Keywords:** extracellular vesicles, virus, diagnosis, biomarker, virus-related diseases

## Abstract

Extracellular vesicles (EVs), a heterogeneous population of lipid bilayer-enclosed membranous particles, are widely involved in cell-cell communication and pathophysiological regulation. Recent advances reveal their dual functionality in viral pathogenesis: while facilitating viral dissemination through transport of pathogenic components, they simultaneously orchestrate host antiviral defense mechanisms. The unique molecular cargo loading capacity and high stability of EVs in body fluids make them ideal biomarkers for early infection diagnosis, treatment monitoring, and prognostic evaluation of virus-related disease. Compared to traditional viral detection methods, EV-based liquid biopsy techniques exhibit distinct advantages, including non-invasiveness and enhanced sensitivity thresholds. This review systematically examines the diagnostic potential of EV biomarkers in viral infections, offering novel perspectives for developing precision diagnostics and therapeutic interventions.

## 1 Introduction

Extracellular vesicles (EVs) have undergone a paradigm shift in biological conceptualization. Prior to the 1980s, academia generally regarded them as “cellular metabolic trash cans”, believing they only served the passive function of expelling metabolic waste (Rashed et al., 2017). Contemporary research has revealed that EVs, a heterogeneous population of lipid bilayer-enclosed nanoparticles, as highly dynamic biological active carrier systems, are ubiquitously produced by both prokaryotic and eukaryotic organisms and actively secrete into the extracellular milieu under conditions of physiological homeostasis or pathological microenvironmental stress, executing sophisticated intercellular communication through the targeted transfer of biomolecular cargos including proteins, lipids, and nucleic acids (Choi et al., 2015). These nano-scale vesicles not only orchestrate essential physiological regulatory networks but also mediate pathogenic mechanisms in various disease states, including viral infection (Kalluri and McAndrews, 2023).

In the intricate interplay between virus and host organisms, EVs exhibit unique biological double-edged sword characteristics: serving as the “Trojan horse” of viral transmission, they assist pathogens in escaping immune surveillance by covertly transporting viral components (Gould et al., 2003); while host cells regulate EV secretion, delivering antiviral immune effector molecules, counteracting viral invasion (Moulin et al., 2023).

While significant progress has been made in the biological functions and diagnostic biomarker potential of EVs in tumorigenesis (Li et al., 2023; Manning et al., 2024), their value in viral infectious diseases remains underexplored. Conventional pathogen detection methods are constrained by limitations in sensitivity, specificity, and invasiveness, creating a clinical urgency to develop EV-based non-invasive diagnostic systems. This review will systematically elaborate on the regulatory role of EVs in various viral infections, focusing on their application prospects and challenges as novel biomarkers in the diagnosis of viral infections.

## 2 Exosome biogenesis

Based on distinct biogenesis pathways, EVs are categorized into three primary subtypes: exosomes, microvesicles, and apoptotic bodies (Cocucci and Meldolesi, 2015). Exosomes, small endosome-derived EVs (30–160 nm, averaging ~100 nm), form via a unique intracellular membrane trafficking process: inward budding of the plasma membrane generates multivesicular bodies (MVBs), which subsequently fuse with the plasma membrane to release intraluminal vesicles (ILVs) into the extracellular space (Mathieu et al., 2019; Kalluri and LeBleu, 2020). Microvesicles (50 nm–1 μm) emerge through direct outward budding of the plasma membrane (Cocucci and Meldolesi, 2015; Mathieu et al., 2019). Apoptotic bodies (1,000–5,000 nm) are generated during programmed cell death (Akers et al., 2013).

Exosome biogenesis is a highly complex and dynamic process, co-regulated by cell type, molecular cargo, and various cellular stimuli, resulting in the generation of highly heterogeneous exosome populations (van Niel et al., 2018). The core mechanism for loading biomolecular cargo into ILVs of multivesicular endosomes relies on the endosomal sorting complex required for transport (ESCRT) machinery (comprising ESCRT-0, -I, -II, -III, and Vps4) and its accessory proteins (such as Alix/PDCd6IP, TSG101, HRS) (Henne et al., 2011). Specifically, ESCRT-0 and ESCRT-I are responsible for clustering ubiquitinated transmembrane cargo into specific microdomains, while ESCRT-II recruits ESCRT-III, which executes membrane deformation and invagination (Hurley, 2008). Disruption of ESCRT components significantly impacts exosomal composition and secretion (Colombo et al., 2013). Additionally, molecules such as syntenin, ALIX, and VPS32 play crucial roles in this process (Baietti et al., 2012).​

In addition to the ESCRT-dependent pathway, ESCRT-independent mechanisms also exist for generating exosomes with specific compositions. One significant pathway is mediated by neutral sphingomyelinase (nSMase), which converts sphingomyelin to ceramide. The production of ceramide can induce the formation of specific microdomains on membranes that promote negative membrane curvature, facilitating budding. Ceramide can be further converted to sphingosine-1-phosphate, activating receptors crucial for cargo sorting (Trajkovic et al., 2008; Goñi and Alonso, 2009). The tetraspanin family of proteins (such as CD81, CD83, CD9, and CD63) also regulates biogenesis through non-ESCRT mechanisms; they cluster and, together with other transmembrane proteins and cytoplasmic proteins within membrane microdomains, induce membrane budding (Charrin et al., 2014). Furthermore, the type of cargo itself influences sorting: transmembrane cargo relies on endosomal machinery, while GPI-anchored proteins may alter membrane properties through their affinity for lipid rafts and participate in budding (de Gassart et al., 2003). Soluble proteins can be packaged within ILVs by being co-sorted with chaperone proteins (like HSP70, HSC70) present in exosomes (Géminard et al., 2004).

**Figure 1:**
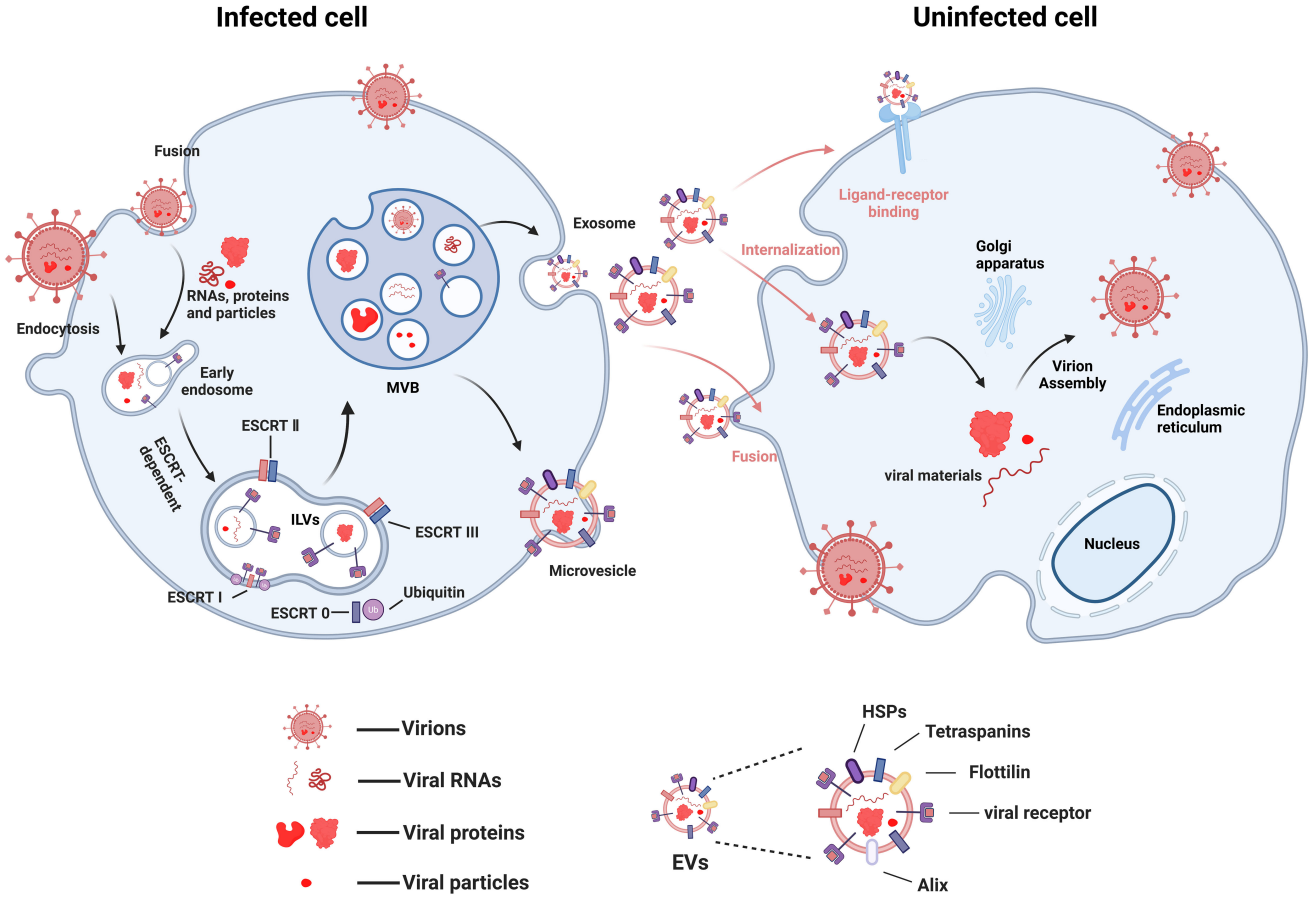
Possible mechanisms of virus transmission using EVs (figure created by BioRender).

Viral infection disrupts the normal physiological processes of host cells, including metabolism and the reorganization of the endomembrane system. This inevitably interferes with fundamental cellular processes related to exosome biogenesis. Many viruses exploit endocytic entry into cells and hijack the exosomal pathway to facilitate their own replication and pathogenesis (Reyes-Ruiz et al., 2020; Velázquez-Cervantes et al., 2023) (**Figure 1**). For instance, viruses such as West Nile virus (WNV) (Chu and Ng, 2004), Zika virus (ZIKV) (Zhou et al., 2018), and Dengue virus (DENV) (Piccini et al., 2015) enter cells via clathrin- or receptor-mediated endocytosis, and their life cycles involve the endosomal/exosomal pathway. Human Immunodeficiency Virus (HIV) serves as another prominent example, being highly dependent on the endosomal/exosomal system for its replication. Exosomes and HIV virions exhibit significant similarities in their biogenesis, biophysical/molecular properties, and cellular uptake mechanisms (Dias et al., 2018). Based on this resemblance, the “Trojan exosome hypothesis” proposes that HIV may directly utilize the exosome system to infect target cells, potentially even bypassing the classical interaction mechanism involving its envelope proteins and cellular receptors (Gould et al., 2003).

## 3 New EV subtypes

**Figure 2:**
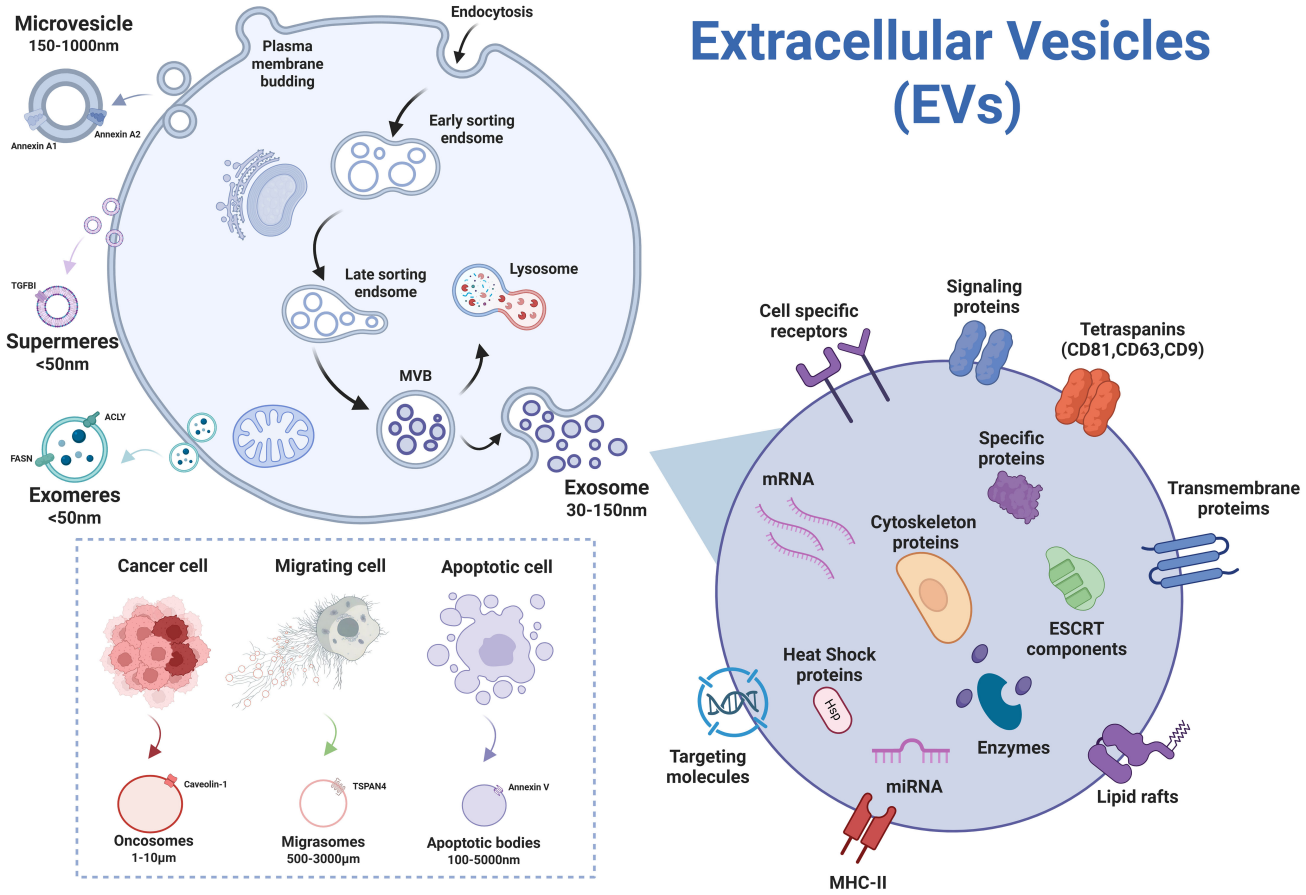
Subtypes of EVs secreted by cells (figure created by BioRender).

Recent years have witnessed the identification of novel EV subtypes through rapid advancements in the field, including oncosomes (Minciacchi et al., 2015; Meehan et al., 2016), migrasomes (Ma et al., 2015; Zhang et al., 2022), exomeres (Zhang et al., 2018; Zhang Q. et al., 2019), and supermeres (Zhang et al., 2021). **Figure 2** illustrates the subtypes of EVs and their different biogenetic mechanisms. Oncosomes, tumor-specifically secreted large-scale vesicles (up to 10 μm), are characterized by surface enrichment of tumor-associated markers and intravesicular cargo containing oncogenic non-coding RNAs, DNA fragments, and pro-angiogenic factors, demonstrating pivotal roles in tumor metastasis and microenvironmental regulation (Minciacchi et al., 2015). Migrasomes, formed through membrane protrusions at retraction fiber tips during cell migration, feature TSPAN4 proteins that coordinate with chemokines to establish spatial positioning signals guiding subsequent migratory trajectories (Ma et al., 2015). Exomeres and Supermeres, recently identified subtypes of EVs, lack a traditional lipid bilayer structure and primarily carry protein and nucleic acid components. Exomeres(<50nm), first isolated and characterized in 2018 via asymmetric flow field-flow fractionation (AF4), exhibiting enrichment in metabolism-associated proteins but lacking canonical exosome markers (e.g., endosomal membrane proteins and ESCRT complex components), suggesting their potential formation through non-classical secretory pathways (Zhang et al., 2018; Yu et al., 2025). Subsequent isolation of smaller nanoparticles termed Supermeres (25–35 nm) from the Exomere-depleted supernatant, which carry disease-related proteins, likely originating from shedding of cell surface molecular fragments or release of cytoplasmic complexes (Clancy et al., 2021; Jeppesen et al., 2022). Their precise biogenesis mechanisms remain incompletely elucidated, and researchers are working to track the origins and pathways of exomeres and supermeres within cells, which can deepen our understanding of intercellular communication mechanisms and lay the foundation for the application of these particles in diseases.

Since the biogenesis pathways differ among subtypes of EVs, their distinct characteristics, including size and molecular markers, are utilized for subtype discrimination (**Table 1**). However, owing to the technical limitations of current isolation methods in achieving precise subtype separation based on biogenesis (van Niel et al., 2018), the International Society for Extracellular Vesicles (ISEV) recommends standardized nomenclature using size-based terms—small EVs (sEVs, <200nm) and large EVs (lEVs, >200nm)—unless rigorous validation of biogenetic origin and phenotypic characterization has been performed (Welsh et al., 2024). Currently, significant knowledge gaps persist regarding their biogenesis pathways and intercellular communication mechanisms, particularly concerning cargo sorting specificity, membrane composition dynamics, and functional heterogeneity across physiological and pathological contexts, necessitating comprehensive mechanistic investigations.

**Table 1:** Different types of EVs.

EV subtypes	Diameter (nm)	Origin	Membrane	Biomarkers	Origin mechanism	Refs.
Exosomes	30-150	MVB (endosomal pathway)	Lipid bilayer	CD9, CD63, CD81, Tsg101, CD81, ALIX, HSP70	MVB fuses with the cell membrane to release ILVs into the extracellular space, forming exosomes	(Mathieu et al., 2019; Kalluri and LeBleu, 2020)
Microvesicles	100–1000	Plasma membrane	Lipid bilayer	Annexin A1, Annexin A2	Direct plasma membrane budding and cleavage	(Cocucci and Meldolesi, 2015; Mathieu et al., 2019)
Apoptotic Bodies	100-5000	Apoptosis cell	Lipid bilayer	Annexin V	Programmed cell death-induced membrane blebbing and fragmentation, packaging cytoplasmic components	(Akers et al., 2013)
Oncosomes	1000–10000	the shedding of non-apoptotic plasma membrane blebbing	Lipid bilayer	Caveolin-1	Membrane budding or cytoplasmic shedding during the malignant transformation of tumor cells	(Minciacchi et al., 2015; Meehan et al., 2016)
Migrasomes	500-3000	Bifurcation of Retraction fibers, tetraspanin-enriched macro domains	Lipid bilayer	TSPAN4	Mechanochemical signaling-driven membrane protrusion rupture during cell migration	(Ma et al., 2015; Zhang et al., 2022)
Exomeres	≤50	Secreting from cells	membraneless	FASN,ACLY	Unknown	(Zhang et al., 2018; Zhang Q. et al., 2019)
Supermeres	~ 35, < 50	Unknown	membraneless	TGFBI	Unknown	(Zhang et al., 2021)

## 4 Diagnostic potential of EVs

As natural intercellular messengers, EVs selectively package bioactive molecules including proteins, nucleic acids, and lipids, with superior molecular abundance and targeting specificity over traditional biomarkers. EVs have emerged as a cutting-edge research focus for next-generation disease biomarkers due to their unique biological properties. Their characteristic phospholipid bilayer structure confers exceptional stability in biofluids, enabling robust detection in blood (Caby et al., 2005), saliva (Palanisamy et al., 2010), urine (Pisitkun et al., 2004), and breast milk (Kosaka et al., 2010). EV-based liquid biopsies offer minimally or non-invasive diagnostic alternatives that are less painful, stress-free, and cost-effective compared to conventional techniques (Vlassov et al., 2012). Crucially, EVs shield their cargo from biofluid interference, providing a more accurate reflection of the pathophysiological state of their parental cells and serving as a reliable window for dynamic disease monitoring (**Table 2**).

**Table 2:** Summary of the diagnostic potential of EVs in virus-related diseases.

Virus	Molecular in EVs	Specimen provenance	Mechanism	Applications	Ref.
HIV	Quantity of EVs in CSF	CSF specimens (n=190; HIV+ on cART: n=67, HIV-: n=45)	The concentration of EVs in the CSF of HIV-positive patients with neurocognitive impairment was significantly increased and shows a strong positive correlation with the neuroinjury marker NFL.	Potential biomarker of HIV-associated central nervous system injury	(Guha et al., 2019)
	HMGB1, NFL, and Aβ	HIV+ on ART (n=23): Mild cognitive impairment (n=11), HIV- (n=12): Mild cognitive impairment (n=3)	The combined detection of HMGB1, NFL, and Aβ in plasma NDEs can specifically distinguish individuals with cognitive impairment.	Reflect the pathological state of neurons and distinguishing HIV-related cognitive impairment	(Sun et al., 2017)
	Nef	Elderly HIV patients on cART: (n=21): HAD (n=12), normal cognition (n=9)	Nef is present in plasma EVs from HAD patients and could significantly increase the Aβ1–42 secretion level of SH-SY5Y nerve cells.	Potential biomarker and therapeutic targets of HAD	(Khan et al., 2016)
	Abundance, size, and microRNA cargo of EVs​	HIV+: n=43 (17 ART-naive, 13 ART-treated with undetectable viral load, and 13 elite controllers), HIV-: n=16	ART-naive HIV-1 infected individuals have significantly elevated plasma concentrations of AChE+ EVs, with larger average particle sizes, and are significantly correlated with immune damage indicators.	Reflect the viral replication status and degree of immune impairment of HIV-1 infection	(Hubert et al., 2015)
EBV	miR-BART7-3p	NPC xenograft model and clinical specimens (NPC: n=5, tumor controls: n=2, EBV carriers: n=3, healthy control: n=1)	Plasma EV-miR-BART7- 3p levels in untreated NPC patients were significantly higher than those in healthy EBV carriers and non-NPC tumor patients, and BART miRNAs could still be detected in EVs even if EBV DNA was not detected in plasma of some patients.	Potential biomark of NPC	(Gourzones et al., 2010)
	miR-BART13-3p	Healthy controls: n=33, NPC: n=39, tumor controls: n=29	EVs actively secreted by EBV-positive NPC cells selectively enriched for viral miRNA BART13-3p. In the serum of patients, the level of EV-BART13-3p was significantly elevated and directly correlated with the presence of tumors.	Potential biomark of NPC	(Ramayanti et al., 2019)
	miR-134-5p, miR-205-5p, miR-409-3p	Healthy controls: n=40, NPC: n=60	The triple diagnostic model based on EV-miR-134- 5p, miR-205- 5p and miR-409- 3p not only distinguishes EBV infection status (positive/negative), but also effectively discriminates early (stage I-II) and late (stage III-IVa) NPC patients.	Alternative or supplementary methods for NPC diagnosis	(Jiang et al., 2021)
	CYPA	Healthy controls: n=36, NPC: n=110	The expression level of CYPA in the serum and EVs of patients with NPC was significantly increased, and EBV-positive cancer cells secreted significantly higher levels of EV-CYPA.	CYPA as a potential non-invasive diagnostic biomarker of NPC	(Liu et al., 2019)
HBV	HBV-DNA	HBV+: n=39	HBV-DNA negative serum patients’ EVs can still detect HBV-DNA, with a sensitivity far exceeding the conventional serum detection limit.	Complementary diagnostic tool for latent HBV infection	(Xu et al., 2023)
	miR-21	Healthy controls (n=30), HCC patients (n=30): HBV+ (n=24), HBV- (n=6), CHB patients (n=30): HBV+ (n=22), HBV- (n=8)	Serum EVs-miR-21 expression levels in HCC patients are significantly higher than those in CHB patients and healthy control groups, and their expression intensity is significantly correlated with the presence of cirrhosis and tumor clinical staging.	Potential early warning marker in the progression of chronic hepatitis to cirrhosis and liver cancer	(Wang et al., 2014)
	miR-212	Healthy controls: n=70, HBV-infected patients: n=200, HBV-infected primary liver cancer patients: n=31	EV-miR-212 expression in HBV-infected patients is significantly higher than in the healthy control group, particularly showing specific elevation in patients with HBV-related liver cancer. Compared to traditional markers, EV-miR-212 demonstrates superior performance in terms of diagnostic sensitivity and specificity.	Indicators for monitoring the progression of HBV-related liver disease	(Zhang Y. et al., 2019)
	NEAT1	ACHBLF patients: n=185	Serum EV-NEAT1 levels in ACHBLF patients were significantly elevated in 90-day non-survivors, with superior predictive efficacy compared to traditional MELD scores.	Potential biomarker for prognostic assessment in ACHBLF patients	(Gao et al., 2021)
HCV	CD81	CHC patients: n=37, patients with chronic HCV infection and normal ALT levels: n=24, long-term sustained virologic response patients: n=7, healthy controls: n=23	Chronic HCV-infected patients show significantly elevated serum EV-CD81 levels, particularly in untreated or persistently infected populations. Patients with chronic HCV infection and persistently normal ALT levels, and patients with long-term SVR, had similar soluble CD81 levels as healthy controls.	Dynamic assessment indicators of liver inflammation and fibrosis	(Welker et al., 2012)
	miR-122-5p,miR-92a-3p, miR-3615, miR-423-3p, miR-128-3p	CHC patients (HCV+:n=21, HCV+/HIV+:n=29)	In CHC patients, EV-miR-122-5p and EV-miR-92a-3p combination can effectively distinguish liver fibrosis (AUC = 0.833). In the HCV mono-infection group, EV-miR-3615 is highly correlated with fibrosis progression (AUC = 0.936), while in the HCV and HIV co-infection group, EV-miR-423-3p and EV-miR-128-3p serve as specific markers (AUC > 0.9).	Dynamic monitoring of liver fibrosis progression	(Cairoli et al., 2025)
	miR-122	Healthy controls: n=16, HepC 1b patients: n=47	The level of EV-miR-122 in HCV genotype 1b patients is negatively correlated with viral load and can predict the effectiveness of antiviral therapy.	Biomarkers of antiviral therapy efficacy and potential therapeutic targets	(Jiao et al., 2017)
HPV	HPV16 DNA	Saliva specimens from patients with HPV-driven OPC (HPV p16+: n=10, healthy controls: n=20)	In HPV-positive oropharyngeal cancer patients, the detection rate of HPV16 DNA in EVs derived from saliva can reach 80%, and the expression levels of glycolysis-related enzymes carried by them are significantly upregulated, which could regulates glucose metabolism and drive OPC.	Potential biomarker of HPV-associated oropharyngeal cancer	(Tang et al., 2021)
	miR-205-5p, miR-1972	HPV+ cell lines (UD-SCC-2, UM-SCC47, UPCI: SCC90), HPV- cell lines (PCI-13 and PCI-30)	EVs derived from HPV-positive HNC cells specifically carry oncogenic mRNA of E6/E7 and miR-205-5p, while EVs from HPV-negative cells show significant enrichment of miR-1972.	Potential biomarker of HPV 16(+)- HNC	(Ludwig et al., 2020)
	HOTAIR, MALAT1, MEG3	Cancer-free normal subjects(n=60; HPV-: n=30, HPV+: n=30), Cervical cancer patients (n=30)	EVs from cervicovaginal lavage fluid of cervical cancer patients exhibited significant upregulation of HOTAIR and MALAT1, with concomitant downregulation of MEG3. The lncRNA expression levels in EVs from HPV-positive non-cancer individuals fall between those of cervical cancer patients and the HPV-negative control group.	Early Identify cervical cancer patients	(Zhang et al., 2016)
SARS-CoV-2	PLT-EVs count	Sars-Cov-2-: n=62, Sars-Cov-2+: n=69	The PLT-EVs count in peripheral blood is significantly elevated in COVID-19 patients.	A diagnostic biomarker of SARS-CovV-2 infection	(Cappellano et al., 2021)
	GM3	Healthy controls (n=26), COVID-19 patients of differing disease severity (mild, moderate, and severe): n=50	The enrichment level of GM3 in plasma EVs is positively correlated with COVID-19 progression.	Assessing the progress and severity of COVID-19	(Song et al., 2020)
	miR-7-5p, miR-24-3p, miR-145-5p, miR-223-3p	Young group (n = 15, age < 30), Elderly group (n = 15, age > 60); Healthy group (n = 15), Diabetic group (n = 15)	Ev-miRNAs (miR-7-5p, miR-24-3p, miR-145-5p and miR-223-3p) are remarkably increased in the young group and healthy group, which could directly inhibit SARS-CoV-2 replication *in vitro*.	SARS-CoV-2 susceptibility assessment and potential therapeutic strategies	(Wang et al., 2021)
ZIKV	Particle size of EV, EV cargo	Macaque trophoblast stem cell model (TSCs, STs)	Physical properties of EVs (average particle size increases) and molecular cargo (including mRNA, miRNA, and protein profiles) undergo specific changes after ZIKV infection, reflecting the infection status and immune response of placental cells.	Non-invasive biomarkers of placental infection	(Block et al., 2022)
DENV	miR-96-5p, miR-146a-5p	Serum specimens (Dengue fever patients: n=272, healthy controls: n=43)	The expression level of miR-96-5p was significantly upregulated as the disease progressed towards severe dengue, while miR-146a-5p expression was markedly downregulated, strongly correlating with thrombocytopenia.	Biomarkers to diagnose and predict dengue disease progression	(Pradhan et al., 2023)
RV	TN-C	Asthma patients and non-asthma primary bronchial epithelial cells	The TN-C content of EVs in BALF from RV-induced asthma patients is positively correlated with the severity of the disease.	Evaluating biomarker for RV-induced acute asthma exacerbations	(Mills et al., 2019)
BK virus	miR-B1-5p/3p	Urine specimens (BKVN patients: n=13, kidney transplant recipients: n=445)	Upregulated expression in urine EVs of BKVN patients.	Possible diagnostic marker for BKVN	(Kim et al., 2017)

In virological research, EVs have been demonstrated to transport viral-specific components including genomic RNA and envelope proteins alongside host stress biomarkers such as pathogen-associated microRNAs (miRNAs) during infection progression (Pegtel et al., 2010; Choi et al., 2013). These virus-associated EVs exhibit early detection advantages in biofluids prior to seroconversion or detectable viremia in conventional diagnostic assays (Liu et al., 2019; Hu et al., 2022). In addition, changes in the dynamic composition of EVs can assess antiviral treatment effectiveness or latent infection activation (Welch et al., 2019), opening new avenues for pathogen detection and host immune response monitoring.

### 4.1 HIV

Despite significant progress in antiretroviral therapy(ART), HIV-associated neurocognitive disorder (HAND) remains a complex neurological complication affecting approximately 50% of HIV-infected individuals. Although numerous screening tests for HAND diagnosis exist, improved methods for early identification and tracking require further development (Saylor et al., 2016). Contemporary research has revealed that EVs exhibit substantial potential as multifaceted biomarkers for both diagnostic and therapeutic applications in HAND. Cerebrospinal fluid (CSF) from HIV-positive on combined anti-retroviral therapy (cART) individuals has been found to contain markedly elevated EV concentrations relative to healthy controls, with particularly pronounced enrichment observed in patients with HAND. This elevated EV abundance demonstrates a robust positive correlation with the neuronal injury biomarker neurofilament light chain (NFL). Longitudinal analytical studies have corroborated that temporal fluctuations in EV concentration closely parallel both NFL dynamics and progressive neurocognitive impairment, thereby suggesting their potential utility as innovative biomarkers for monitoring central nervous system injury progression (Guha et al., 2019).

In contrast to the invasive lumbar puncture required to obtain CSF, plasma EVs can be collected non-invasively via routine blood sampling, greatly enhancing their practical utility. Investigations into plasma neuron-derived EVs (NDEs) reveal that the enrichment of neuron-specific proteins carried by NDEs isolated by the neuronal cell adhesion molecule L1CAM antibody immunoprecipitation demonstrates diagnostic efficacy in quantifying neuronal injury severity. NDEs from cognitively impaired patients exhibit significant elevations in high-mobility group protein B1 (HMGB1), NFL, and β-amyloid (Aβ) concentrations (Sun et al., 2017). Another study revealed that EVs containing both Nef protein and Nef mRNA in HIV-positive on cART individuals exhibit neuroinvasive potential, demonstrating capacity for blood-brain barrier (BBB) transmigration and subsequent neuronal internalization. *In vitro* experiments utilizing SH-SY5Y neural cells demonstrated that EVs isolated from plasma of HIV-associated dementia (HAD) patients significantly upregulated Aβ1–42 secretion, exhibiting a strong positive correlation with EVs-Nef protein load, suggesting that EVs-Nef might serve as potential biomarkers for HAD (Khan et al., 2016).

Plasma EVs from HIV-positive patients may also serve as markers of disease progression, with analyses integrating their physical characteristics and molecular cargo offering novel insights for clinical staging and therapy evaluation.​Plasma-derived acetylcholinesterase-positive (AChE+) EVs from ART-naive HIV-1-infected individuals exhibit increased particle size and elevated abundance, which show significant inverse correlations with CD4/CD8 ratios and positive associations with elevated CD8+ T-cell counts. Notably, the enrichment patterns of miRNAs (miR-155, miR-223) in EVs further indicate their functional involvement: miR-155 correlates with immune activation, while miR-223 is associated with inflammatory regulation. Both miRNAs exhibit significantly negative correlations between their levels in EVs and EV abundance/size in ART-naive patients, reflecting abnormalities in molecular regulatory networks during disease progression (Hubert et al., 2015).

### 4.2 EBV

Epstein-Barr virus (EBV), the first identified oncogenic γ-herpesvirus in humans, has been demonstrated to exhibit a definitive etiological association with the pathogenesis of malignant tumors including nasopharyngeal carcinoma (NPC) (Kimura and Kwong, 2019). Empirical evidence reveals that EVs secreted by EBV-positive NPC cells selectively enrich virally encoded BART miRNAs, such as miR-BART7-3p and miR-BART13-3p (Gourzones et al., 2010; Ramayanti et al., 2019). These viral miRNAs exhibit significant elevation in plasma EVs from NPC patients, demonstrating a positive correlation with tumor progression status. Remarkably, their detectable presence persists even in serologically EBV DNA-negative cases, thereby suggesting clinical potential as complementary diagnostic biomarkers (Gourzones et al., 2010). BART-miRNAs exhibit unique sorting mechanisms within EVs, which not only enhance detection reliability but, more critically, enable precise differentiation between NPC and other head and neck malignancies or latent EBV infections. For instance, miRNA-BART13-3p is specifically enriched within EVs derived from NPC tumor cells, yet is virtually undetectable in EVs from EBV-negative head and neck cancer cells or cells with asymptomatic latent EBV infection. In patient serum samples, EV-associated BART13-3p levels were significantly elevated and showed a direct correlation with tumor presence. Its diagnostic performance outperformed conventional plasma EBV DNA load testing and EBNA1 IgA serological methods (Ramayanti et al., 2019).

Beyond virally encoded components, host-derived EV-associated molecules also hold critical diagnostic significance. Through comparative profiling of plasma EV-miRNA expression between NPC patients and healthy controls, followed by multi-stage screening and validation, researchers constructed a diagnostic model based on three specific EV-miRNAs (miR-134-5p, miR-205-5p, and miR-409-3p). This diagnostic model demonstrated excellent discriminatory power in both the training set (AUC=0.88) and the validation set (AUC=0.91), exhibiting outstanding diagnostic and staging capabilities for distinguishing EBV infection status as well as early (stage I-II) and late (stage III-IVa) NPC (Jiang et al., 2021). Furthermore, enrichment of cyclophilin A (CYP-A) in serum EVs from EBV-associated NPC patients showed significantly enhanced diagnostic performance (AUC=0.844) compared to whole-serum assays. Importantly, this biomarker effectively compensated for the suboptimal sensitivity of conventional EBV-VCA-IgA testing in seronegative subgroups, highlighting EVs’ unique advantage in NPC diagnostics (Liu et al., 2019).

### 4.3 Hepatitis virus

Hepatitis virus infections constitute a primary global driver of liver fibrosis, cirrhosis, and hepatocellular carcinoma (HCC). EVs have emerged as breakthrough biomarkers in the diagnosis and management of viral hepatitis-related liver diseases. In hepatitis B virus (HBV) infection, EV-encapsulated HBV-DNA overcomes the sensitivity limitations of serological assays, detecting high viral loads in chronic HBV patients with undetectable serum HBV-DNA, thereby providing critical molecular evidence for occult HBV infection (Xu et al., 2023). Beyond diagnosis, EV-associated non-coding RNAs show significant promise for monitoring the progression of HBV-related liver disease. Specifically, serum EV levels of miR-21 are significantly elevated in hepatocellular carcinoma (HCC) patients compared to those with chronic hepatitis, and this overexpression correlates with both fibrosis progression and tumor stage, demonstrating diagnostic value that surpasses whole-serum biomarker analysis (Wang et al., 2014). Similarly, EV-miR-212 shows disease specific overexpression in HBV-associated HCC, exhibiting positive correlations with clinical indicators such as the Child-Pugh and Model for End-stage Liver Disease (MELD) score, while surpassing conventional tumor markers AFP, CA125 and HBx in diagnostic accuracy (Zhang Y. et al., 2019). Furthermore, EVs emerge as vital prognostic​​ biomarkers in acute liver failure phases. Notably, the level of the long non-coding RNA (lncRNA) NEAT1 within serum EVs serves as a significant and independent predictor of 90-day mortality in acute-on-chronic hepatitis B liver failure (ACHBLF) patients. Non-survivors present significantly higher serum EV NEAT1 levels than survivors. Importantly, for predicting 90-day mortality, serum EV NEAT1 outperforms the widely-used MELD scoring system in diagnostic performance (Gao et al., 2021).

In the pathogenesis of hepatitis C virus (HCV), the tetra-span membrane protein CD81 is the first host factor reported to interact with the soluble form of HCV glycoprotein E2, and is essential for HCV infection of liver cells (Pileri et al., 1998; Fénéant et al., 2014). As a key host receptor, CD81 mediates viral entry into liver cells, and its expression dynamics are closely related to disease progression (Masciopinto et al., 2004). Chronic HCV (CHC) carriers demonstrate elevated serum EV-associated CD81 levels, particularly pronounced in treatment-naive or persistently infected individuals, with expression positively correlating with hepatic injury markers (alanine aminotransferase [ALT]) and fibrosis staging. In chronic HCV infected individuals with persistently normal ALT levels, as well as in those who have long-term sustained virologic response (SVR), serum EV-associated CD81 levels are similar to those of the healthy control group, suggesting its utility as a dynamic biomarker for monitoring hepatic inflammation and fibrogenesis (Welker et al., 2012). Regarding diagnostic applications, EV-derived miRNA profiling reveals that combinatorial analysis of miR-122-5p and miR-92a-3p effectively discriminates clinically significant fibrosis (F≥2) in CHC patients, demonstrating superior diagnostic performance compared to conventional indices including the aspartate aminotransferase-to-platelet ratio index (APRI) and Fibrosis-4 (FIB-4) scoring system. Notably, distinct EV-miRNA signatures emerge between HCV mono-infection and HCV/HIV co-infection cohorts: miR-3615 shows strong fibrosis-stage association in mono-infected patients, while co-infected individuals exhibit unique signatures marked by miR-423-3p and miR-128-3p overexpression (Cairoli et al., 2025).

Building upon the critical role of EV-associated molecular signatures for disease stratification, their utility extends powerfully into treatment response prediction, particularly for HCV genotype 1b, which dominates in regions like Asian and exhibits variable interferon-based therapy outcomes. Research revealed that in patients with HCV genotype 1b, both serum and exosomal miR-122 levels demonstrated a significant inverse correlation with corresponding HCV RNA viral load. Crucially, miR-122 exhibited substantial predictive utility for SVR to antiviral therapy. Patients achieving SVR displayed significantly higher miR-122 levels compared to non-responders, validated by ROC curve analysis, which yielded an AUC of 0.956 (sensitivity 90.5%, specificity 96.2%), surpassing established parameters like the ALT/AST ratio. Within this cohort, the majority of genotype 1b patients attained SVR, contrasting with the inferior therapeutic efficacy observed in non-genotype 1b (non-1b) patients. The relatively elevated miR-122 expression observed in genotype 1b patients, compared to both healthy controls and non-1b patients, may constitute a key factor contributing to their more favorable treatment outcomes. These findings collectively suggest that miR-122 not only serves as a predictive biomarker for treatment efficacy but also represents a potential therapeutic target for novel antiviral agents (Jiao et al., 2017).

### 4.4 HPV

Human papillomavirus (HPV), a non-enveloped double-stranded DNA virus, is primarily transmitted via sexual contact and infects mucosal epithelia of the anogenital tract, oropharynx, and perianal regions (Wang et al., 2018). Persistent infection with oncogenic HPV types predisposes to the development of tumors in these sites. Recent research underscores the diagnostic potential of EVs in HPV-associated malignancies. Saliva-derived EVs from patients with HPV-driven oropharyngeal cancer (OPC) contain detectable HPV16 DNA and exhibit elevated expression of six key glycolytic pathway enzymes: aldolase (ALDOA), glyceraldehyde-3-phosphate dehydrogenase (GAPDH), lactate dehydrogenase A (LDHA), lactate dehydrogenase B (LDHB), phosphoglycerate kinase 1 (PGK1), and pyruvate kinase M1/2 (PKM). These EV-associated markers demonstrate high diagnostic accuracy in distinguishing HPV-driven OPC from healthy controls (Tang et al., 2021). Head and neck cancers (HNCs), among the most common malignant diseases worldwide, represent another category of HPV-associated cancer. EVs derived from HPV-positive HNC cells uniquely carry E6/E7 oncogene transcripts and miR-205-5p. In contrast, EVs derived from HPV-negative HNC cells exhibit marked enrichment of miR-1972 (Ludwig et al., 2020). The expression levels of these EV-miRNAs depend on HPV status, serving as a high-specificity diagnostic panel for the precise determination of HPV infection status.

Cervical cancer ranks among the most common HPV-associated malignancies. Studies show that the dynamic alterations in the lncRNA expression profiles within EVs derived from cervicovaginal lavage fluid exhibit unique diagnostic potential. Specifically, oncogenic lncRNAs, such as HOTAIR and MALAT1, are significantly upregulated in EVs from cervical cancer patients, while the tumor-suppressive lncRNA MEG3 is significantly downregulated. Notably, the expression levels of these lncRNAs in HPV-positive individuals without cancer lie intermediate between those in HPV-negative individuals and HPV-positive cervical cancer patients. This expression pattern suggests that they may act as molecular mediators facilitating the transition from HPV infection to malignant transformation. A multi-analyte detection model integrating HOTAIR, MALAT1, and MEG3 expression patterns achieves high-precision stratification of cervical cancer patients, HPV-persistent carriers, and healthy controls. This discovery establishes a novel molecular framework for developing non-invasive screening tools based on EV-associated lncRNA signatures in cervicovaginal lavage fluid (Zhang et al., 2016).

### 4.5 SARS-CoV-2

The ongoing global pandemic of Coronavirus Disease 2019 (COVID-19), caused by severe acute respiratory syndrome coronavirus 2 (SARS-CoV-2), has spurred intensive research. Recent studies indicate that circulating levels of platelet-derived EVs (PLT-EVs) are significantly elevated in patients infected with SARS-CoV-2 compared to both non-infected patients and healthy individuals. A flow cytometry (FC) detection method based on dual-marker detection of CD41a and CD31 enables the rapid assessment of intact EVs directly within whole blood samples, requiring no sample pretreatment. Clinical analyses in hospitalized patients demonstrate that PLT-EVs exhibit good diagnostic performance (AUC=0.79) in distinguishing COVID-19 infection (Cappellano et al., 2021). This characteristic positions PLT-EVs as a promising rapid diagnostic biomarker for complementary use alongside nucleic acid testing, facilitating the differential diagnosis of symptomatic patients testing negative by nucleic acid assays.

Although the majority of COVID-19 patients experience mild to moderate symptoms, a subset with pneumonia can deteriorate rapidly into severe respiratory failure. To investigate the association between disease severity and metabolic dysregulation, researchers comprehensively analyzed the plasma lipidome and metabolome of healthy controls and COVID-19 patients stratified by disease severity (mild, moderate, severe) using an integrated approach combining targeted and untargeted tandem mass spectrometry. The findings revealed that disease severity was significantly associated with distinct alterations in specific lipid profiles, characterized by elevated levels of sphingomyelins (SMs) and monosialodihexosylganglioside GM3, alongside decreased levels of diacylglycerols (DAGs). Subsequent analysis of isolated plasma EVs demonstrated that the degree of GM3 enrichment within EVs correlated positively with disease severity and inversely with peripheral blood CD4+ T cell counts, exhibiting outstanding diagnostic specificity for identifying critical illness (Song et al., 2020).

Compared with younger people, elderly individuals and patients with underlying medical conditions such as diabetes are more susceptible to COVID-19 infection and at higher risk of developing severe complications. Systematic analysis of circulating EV-miRNAs in COVID-19 patients of different ages and with diabetes mellitus identifies characteristic downregulation of miR-7-5p, miR-24-3p, miR-145-5p, and miR-223-3p in elderly individuals and diabetic patients, with reduced expression strongly correlating with enhanced replication activity of SARS-CoV-2. Mechanistic investigations demonstrate that these miRNAs directly inhibit viral genomic replication by targeting the spike (S) protein-encoding gene, while regular exercise intervention significantly upregulates their EV-enriched expression levels, potentiating vesicle-mediated antiviral host defense (Wang et al., 2021). The dynamic changes of EV-miRNAs underscore their dual potential as tunable therapeutic targets and potential biomarkers to assess COVID-19 susceptibility.

### 4.6 ZIKV

ZIKV infection is closely associated with adverse pregnancy outcomes at the maternal-fetal interface. A study utilizing a macaque trophoblast stem cell (TSC) model demonstrated that TSCs and syncytiotrophoblast-differentiated cells (ST3Ds), exhibit high susceptibility to ZIKV. This susceptibility is characterized by robust viral replication and significant intracellular expression of viral E and NS2B proteins. In contrast, extravillous trophoblasts (EVTs) display relative resistance to infection. Notably, ZIKV infection specifically altered the physical characteristics (such as an increase in the average particle size) and molecular cargo (including mRNA, miRNA, and protein profiles) of EVs secreted by these cells. Mass spectrometry analysis confirmed that the enrichment of ZIKV proteins within EVs directly correlates with the viral replicative state. Furthermore, EVs released from infected syncytiotrophoblast cells exhibited an upregulation in the expression of genes and proteins associated with inflammatory cytokines (e.g., CXCL11) and antiviral pathways. These alterations not only reflect the infection status and immune responses of placental cells but also indicate the potential of EVs to serve as “liquid biopsy” vehicles. Analyzing ZIKV proteins or differentially expressed molecules within placental-derived EVs isolated from maternal blood could provide critical diagnostic insights into ZIKV vertical transmission and placental dysfunction (Block et al., 2022).

### 4.7 DENV

Dengue fever is an acute mosquito-borne infectious disease caused by DENV. As no specific antiviral treatments currently exist, identifying early biomarkers associated with disease progression is crucial for clinical management. An in-depth study of an Indian patient cohort revealed significant and dynamic alterations in the miRNA expression profile carried by serum EVs across different severity stages of dengue. Specifically, the expression level of miR-96-5p was significantly upregulated as the disease progressed towards severe dengue, while miR-146a-5p expression was markedly downregulated, this aberrant miRNA expression pattern was detectable on the first day of development from mild dengue to dengue with warning symptoms. These changes preceded the significant deterioration of traditional clinical indicators such as declining platelet counts, highlighting their potential as early warning biomarkers. Importantly, the study also found a strong positive correlation between the expression levels of both miRNAs and patient platelet counts, further supporting their close association with the pathophysiological processes of the disease. ROC curve analysis demonstrated that miR-146a-5p achieved an exceptionally high AUC of 100% for distinguishing disease progression, while miR-96-5p also attained an AUC of 73.6%. These results indicate their potential as highly specific and accurate non-invasive biomarkers (Pradhan et al., 2023).

### 4.8 Others

Respiratory virus infection studies demonstrate that human rhinovirus (RV) infection triggers bronchial epithelial cells to release pro-inflammatory extracellular matrix protein tenascin-C (TN-C) and EVs. EVs not only serve as functional delivery vehicles for TN-C but also significantly potentiate the secretion of key inflammatory mediators including IL-8, IL-6, and CCL5 by macrophages and airway epithelial cells through Toll-like receptor 4 (TLR4)-independent signaling pathways, thereby playing a central pro-inflammatory role in viral-induced asthma exacerbations. Further research identified EV-TN-C in bronchoalveolar lavage fluid (BALF) from asthma patients shows significant positive correlations with clinical asthma severity (Mills et al., 2019). Given EVs’ pivotal role in amplifying inflammatory cascades and their targeted delivery properties within the airway microenvironment, these bioactive nanoparticles may emerge as novel diagnostic markers for assessing RV-triggered acute asthma episodes.

BK virus nephropathy (BKVN), a transplant-associated complication triggered by BK polyomavirus reactivation (BKV), drives progressive renal allograft dysfunction and graft failure (Borriello et al., 2022). Research reveals specific enrichment of virus-encoded miRNAs (BKV-miR-B1-5p/3p) in urinary EVs from BKVN patients, demonstrating characteristic overexpression during active viral replication phases. When normalized against endogenous control miR-16, this viral miRNA biomarker exhibits superior sensitivity and specificity over conventional urinary BKV-DNA load quantification, providing precise assessment of intrarenal viral activity (Kim et al., 2017). This breakthrough not only provides non-invasive diagnostic strategies for BKVN but also establishes a novel paradigm for precision monitoring of organ-specific viral infections after organ transplantation.

## 5 Challenges

EVs as core mediators of intercellular communication, their clinical application potential and challenges coexist. Although research on EVs as clinical biomarkers has made significant progress, there are still many issues to be resolved (Sanz-Ros et al., 2023).

The isolation of EVs necessitates highly specialized operational protocols, yet current methodologies lack standardized technical criteria. Multiple EV isolation and purification techniques have emerged, with traditional approaches relying on centrifugation technologies such as ultracentrifugation and density-based gradient centrifugation that exploit EVs’ buoyant density characteristics. Subsequent methodologies leverage differential solubility and/or aggregation properties in specific media, exemplified by polyethylene glycol (PEG) precipitation. Recent innovations include immunocapture techniques targeting EV surface molecular affinities and microfluidic-based precision separation devices, which enhance isolation specificity and efficiency. However, persistent limitations involve potential compromise of EV structural and functional integrity: ultracentrifugation subjects vesicles to mechanical stress from high centrifugal forces (Nordin et al., 2015), membrane filtration induces physical deformation through compressive forces (Taylor and Shah, 2015), while immunoaffnity methods employing non-physiological antibodies, extreme pH conditions, or elution buffers may alternative biological properties (Yang et al., 2020).

EVs exhibit substantial heterogeneity and cargo diversity, which introduces notable complexity into analytical processes (Kalluri and LeBleu, 2020). Clinical investigations have demonstrated that patient-derived EVs frequently transport disease-specific biomarkers and pathological components such as pro-inflammatory factors, whereas EVs from healthy individuals predominantly have protective functions such as anti-inflammatory and antioxidant (Skuratovskaia et al., 2021). Distinct EV subpopulations exert differential regulatory effects on recipient cells. For instance, small and large EVs secreted by immature dendritic cells selectively induce Th2 and Th1 cytokine secretion, respectively (Tkach et al., 2017). This functional differentiation likely originates from variations in EV biogenesis pathways and the highly selective cargo-loading mechanisms (Kalluri and LeBleu, 2020). Current EV subtype classification primarily relies on biophysical parameters and molecular signatures (Théry et al., 2018). A proteomic analysis reveals that EVs derived from single-cell sources may contain thousands of protein signals, with numerous uncharacterized subtypes remaining to be systematically elucidated (Choi et al., 2019).

Currently, numerous ongoing clinical trials aim to develop EVs as novel biomarkers (Ghodasara et al., 2023). However, their clinical implementation requires extensive systematic validation studies to achieve successful translation from laboratory research to routine clinical testing.

## 6 Conclusion

EVs as emerging biomarkers for diagnosis and therapeutic monitoring of virus-related diseases, demonstrating significant clinical translational potential. Their unique phospholipid bilayer structure and stable body fluid distribution enable specific encapsulation of viral nucleic acids, proteins, and host stress molecules, mirroring disease progression with high fidelity. In HIV-associated neurocognitive disorders, EV-enriched neuronal proteins (e.g., HMGB1, NFL) and viral components (e.g., Nef) in CSF and plasma enable non-invasive monitoring of CNS injury. For EBV-driven nasopharyngeal carcinoma, EV-packaged viral miRNAs (miR-BART13-3p) and host proteins (CYPA) achieve superior sensitivity over conventional EBV-DNA testing, particularly in seronegative cases. Hepatitis virus infections leverage EV-encapsulated HBV-DNA and miRNA signatures (e.g., miR-21, miR-212) to detect occult infections and stratify liver fibrosis stages, while HCV-specific EV markers (CD81, miR-122) predict treatment response. Similarly, HPV-related cancers exhibit EV-carried oncogenic lncRNAs (HOTAIR, MALAT1) in cervicovaginal fluid for early malignancy detection, and SARS-CoV-2 utilizes platelet-derived EV counts and lipid profiles (GM3) to gauge disease severity. This multifaceted diagnostic utility—spanning early detection, prognostic stratification, therapy monitoring, and differential diagnosis—stems from EVs’ unique capacity to concentrate pathognomonic biomolecules while resisting degradation, outperforming traditional methods in sensitivity, specificity, and clinical practicality.

Notably, research reviewed in this review reveals that the diagnostic utility of EVs in virus-related diseases (**Table 1**) is primarily focused on sEVs, represented by exosomes. Their endosomal biogenesis pathway (**Figure 2**) enables selective enrichment of disease-specific cargos (e.g., viral miRNAs, host stress proteins) while maintaining excellent stability in biological fluids. This dual capacity for targeted molecular packaging and resistance to degradation establishes exosomes/sEVs highly valuable for diagnostics.

Nevertheless, the isolation of EVs still lacks standardized protocols, with their inherent heterogeneity compounding analytical challenges in clinical settings. Rigorous systematic validation studies remain imperative for establishing robust and quantifiable biomarkers that meet clinical diagnostic requirements.

Future research endeavors should prioritize the following strategic directions: (1) Developing high-sensitivity and high-specificity techniques for EV isolation and characterization to advance the standardization and uniformity of clinical detection protocols; (2) Elucidating the molecular mechanisms underlying EV-mediated virus-host interactions to delineate their functional dynamics during infectious processes; (3) Implementing artificial intelligence (AI)-driven multi-omics data integration to establish comprehensive EV biomarker system, thereby facilitating data-driven advancements in precision medicine.

In conclusion, EVs, functioning as “cellular messengers,” exhibit bidirectional effects in viral infection, thereby creating novel avenues for their transition from fundamental research to clinical implementation, with the potential to become a core component of next-generation diagnostic tools and treatment strategies.
